# Symbolic kinetic models in python (SKiMpy): intuitive modeling of large-scale biological kinetic models

**DOI:** 10.1093/bioinformatics/btac787

**Published:** 2022-12-10

**Authors:** Daniel R Weilandt, Pierre Salvy, Maria Masid, Georgios Fengos, Robin Denhardt-Erikson, Zhaleh Hosseini, Vassily Hatzimanikatis

**Affiliations:** Laboratory of Computational Systems Biotechnology, École Polytechnique Fédérale de Lausanne (EPFL), Lausanne 1015, Switzerland; Laboratory of Computational Systems Biotechnology, École Polytechnique Fédérale de Lausanne (EPFL), Lausanne 1015, Switzerland; Laboratory of Computational Systems Biotechnology, École Polytechnique Fédérale de Lausanne (EPFL), Lausanne 1015, Switzerland; Laboratory of Computational Systems Biotechnology, École Polytechnique Fédérale de Lausanne (EPFL), Lausanne 1015, Switzerland; Laboratory of Computational Systems Biotechnology, École Polytechnique Fédérale de Lausanne (EPFL), Lausanne 1015, Switzerland; Laboratory of Computational Systems Biotechnology, École Polytechnique Fédérale de Lausanne (EPFL), Lausanne 1015, Switzerland; Laboratory of Computational Systems Biotechnology, École Polytechnique Fédérale de Lausanne (EPFL), Lausanne 1015, Switzerland

## Abstract

**Motivation:**

Large-scale kinetic models are an invaluable tool to understand the dynamic and adaptive responses of biological systems. The development and application of these models have been limited by the availability of computational tools to build and analyze large-scale models efficiently. The toolbox presented here provides the means to implement, parameterize and analyze large-scale kinetic models intuitively and efficiently.

**Results:**

We present a Python package (SKiMpy) bridging this gap by implementing an efficient kinetic modeling toolbox for the semiautomatic generation and analysis of large-scale kinetic models for various biological domains such as signaling, gene expression and metabolism. Furthermore, we demonstrate how this toolbox is used to parameterize kinetic models around a steady-state reference efficiently. Finally, we show how SKiMpy can implement multispecies bioreactor simulations to assess biotechnological processes.

**Availability and implementation:**

The software is available as a Python 3 package on GitHub: https://github.com/EPFL-LCSB/SKiMpy, along with adequate documentation.

**Supplementary information:**

Supplementary data are available at *Bioinformatics* online.

## 1 Introduction

Organisms are complex and adaptive systems, posing a challenge when investigating their response to environmental or genetic perturbations (Kitano, 2002). In this context, large-scale kinetic models are an essential tool to understand how the underlying biochemical reaction networks respond to such perturbations (Chowdhury *et al.*, 2015). However, currently, no modeling framework allows users to build and analyze large-scale kinetic models efficiently. Therefore, we propose a novel Python toolbox that enables the user to semiautomatically reconstruct a kinetic model from a constraint-based model (Salvy *et al.*, 2019). Furthermore, we express the models in terms of symbolic expressions, allowing the straightforward implementation of various analysis methods, for example, numerical integration of the ordinary differential equations (ODEs).

Such numerical analysis requires a set of kinetic parameters describing the individual reaction characteristics. However, as parameters from literature or databases (Schomburg *et al.*, 2013) are collected *in vitro* and often fail to capture the *in vivo* reaction kinetics (Weilandt and Hatzimanikatis, 2019), a series of methods have been developed to infer parameters from phenotypic observations (Gonzalez *et al.*, 2007; Khodayari and Maranas, 2016; Saa and Nielsen, 2016; Wang *et al.*, 2004). To this end, we here present the first open-source implementation of the ORACLE framework to efficiently generate steady-state consistent parameter sets (Chakrabarti *et al.*, 2013; Miskovic and Hatzimanikatis, 2010; Savoglidis *et al.*, 2016; Tokic *et al.*, 2020; Wang *et al.*, 2004).

## 2 Materials and methods

### 2.1 Implementing kinetic models

The system of ODEs describing the kinetics of a biochemical reaction network can be derived directly from the mass balances of the N reactants participating in the M reactions of the network:

(1) $$\frac{dX_{i}}{dt}=\sum_{j=1}^{M}n_{ij}\;\nu_{j}\left(X,p\right),\;\forall \;i=1,\ldots ,N,$$

where $X_{i}$ denotes the concentration of the chemical i, $n_{ij}$ is the stoichiometric coefficient of reactant i in reaction j and $\nu_{j}(X,p)$ is the reaction rate of reaction j as a function of the concentration state variables $X={\left[X_{1},X_{2},\ldots ,\;X_{N}\right]}^{T}$ and K kinetic parameters $p={\left[p_{1},p_{2},\ldots ,\;p_{K}\right]}^{T}$. The functions $\nu_{j}(X,p)$ are the given rate laws of each reaction j. An overview of the implemented rate laws is given in Supplementary Table S1. If the reactants are distributed across multiple cellular compartments, then each reactant’s mass balance is modified according to (for details, see Supplementary Material):

(2) $$\frac{dX_{i}}{dt}=\frac{V_{\mathrm{Cell}}}{V_{i}}\sum_{j=1}^{M}n_{ij}\;\nu_{j}(X,p),\;\forall \;i=1,\ldots ,N,$$

where $V_{\mathrm{Cell}}$ is the overall cell volume and $V_{i}$ is the compartment volume for concentration $X_{i}$.

### 2.2 Efficient steady-state consistent parameterization

To overcome the scarcity of kinetic data, SKiMpy provides the means to infer the parameters efficiently on a large scale by sampling sets of kinetic parameters consistent with steady-state physiology (Miskovic and Hatzimanikatis, 2010; Wang *et al.*, 2004; Wang and Hatzimanikatis, 2006). These parameter sets are then evaluated for local stability, global stability and relaxation time to discard unstable models and models with non-physiological dynamics.

## 3 Usage

**Fig. 1.**
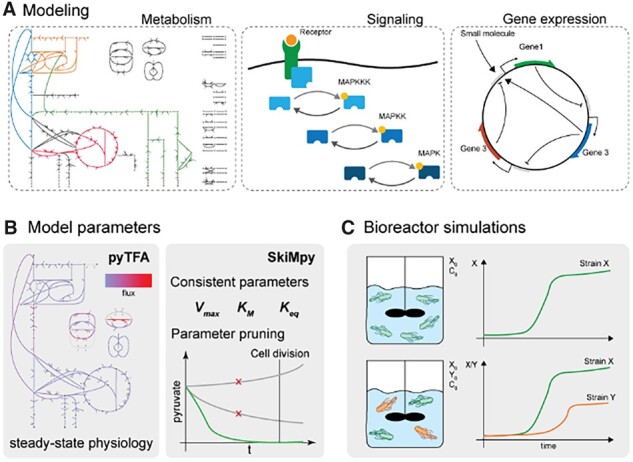
Software capabilities. (**A**) Building and simulation of different types of models including metabolic, signaling and gene expression networks. (**B**) Parameterization of kinetic metabolic models around a reference steady-state derived from constrained-based modeling and parameter pruning based on local stability as well as characteristic time constants and (**C**) bioreactor modeling microbial growth in a dynamic environment for individual and multiple species

SKiMpy enables the user to reconstruct kinetic models for large-scale biochemical reaction systems. With an extensive palette of analytic methods, the software presents a versatile platform to model biological systems, as shown with the examples given in the Supplementary Material with models of (i) *Escherichia coli*’s central metabolism, (ii) a signaling pathway, (iii) synthetic gene-expression circuits and (iv) different strains in a bioreactor (Fig. 1A–C, for details, see Supplementary Material). Furthermore, the software generates symbolic expressions directly available to the user, facilitating method development for large-scale kinetic models.

## 4 Conclusion

Similar to previous work (Dräger *et al.*, 2015; Smith *et al.*, 2018) SKiMpy allows the semi-automated reconstruction of kinetic models from constrained-based models. But instead of focusing on the kinetics of metabolic networks, SKiMpy provides the tools to build integrated models accounting for additional layers of complexity including signaling and gene expression as well as the bioreactor environment. Furthermore, SKiMy implements additional methods to parameterize kinetic models based on physiological observations rather than *in vitro* parameters (Liebermeister and Noor, 2021).

With SKiMpy we present a versatile method development platform to analyze cell dynamics and physiology on a large scale. We believe that the toolbox will facilitate the accessibility of large-scale kinetic models to various biological disciplines and studies ranging from biotechnology to the medical sciences.

## Supplementary Material

btac787_Supplementary_Data
